# Partial Horner’s Syndrome Secondary to Carotid Dissection With an Associated Folate Deficiency

**DOI:** 10.7759/cureus.95254

**Published:** 2025-10-23

**Authors:** Syed Ali Ahmad, Syed Muhammad Meeran Hussain, Komal Makhijani, Yohan Samarasinghe

**Affiliations:** 1 General Medicine, Frimley Park Hospital, Camberley, GBR; 2 Acute Medicine, Frimley Park Hospital, Camberley, GBR

**Keywords:** folate deficiency, horner’s syndrome, hyperhomocysteinemia (hhcy), non-traumatic carotid artery dissection, partial horner’s syndrome, young adult female

## Abstract

Horner’s syndrome is an uncommon clinical finding characterized by the triad of ptosis, miosis, and anhidrosis. It typically results from disruption along the oculosympathetic pathway. When anhidrosis is absent, the partial Horner’s syndrome suggests a lesion distal to the superior cervical ganglion and is an important clinical clue. One of the most important causes in young patients is carotid artery dissection, a potentially serious but often under-recognized condition. We report the case of a 34-year-old woman who presented with a left-sided headache and new-onset ptosis. Examination revealed a partial Horner’s syndrome. Initial blood tests, CT of the head, and chest X-ray were unremarkable, but CT angiography demonstrated a left internal carotid artery dissection with near-occlusion. She was treated with dual antiplatelet therapy. Further investigations into the cause revealed a folate deficiency and mild hyperhomocysteinemia. This case emphasizes the diagnostic value of recognizing partial Horner’s syndrome in localizing the lesion and the importance of urgent vascular imaging to rule out a carotid pathology, especially in a young patient. Additionally, while carotid dissections are often spontaneous or trauma-related, nutritional deficiencies such as folate deficiency with resulting hyperhomocysteinemia represent potentially modifiable risk factors. Early recognition and a comprehensive stroke workup are essential to guide management and reduce the risk of a recurrence.

## Introduction

Horner’s syndrome is a rare condition that usually presents unilaterally, with the classic triad of ptosis (drooping of the eyelid), miosis (constricted pupils), and anhidrosis (loss of sweating) [1]. This occurs as a result of damage to the oculosympathetic pathway, part of the sympathetic system, which consists of three parts. The first-order, or central, neuron originates at the hypothalamus and descends down to intermediolateral cells of the spinal cord, at the C8-T2 level, where it terminates. The second-order, or pre-ganglionic, neuron originates from where the first-order neuron terminates, exiting the spinal cord via the dorsal root, ascending via the cervical sympathetic chain, and synapsing at the superior cervical ganglion. Finally, the third order, or post-ganglionic, neuron originates from the superior cervical ganglion, and most of the fibers travel with the internal carotid artery to the cavernous sinus. There, it joins the ophthalmic division of the trigeminal nerve to reach the orbit and then innervates the iris dilator muscle and Müller muscle, responsible for pupil dilation and eyelid elevation, respectively. The remaining fibers travel with the external carotid artery and its branches to ultimately provide sudomotor (sweat) and vasomotor (blood vessel) innervation to the face [2].

While it may be congenital or hereditary, the vast majority of cases are acquired, and the etiology includes trauma, stroke, infections, tumors, autoimmune, and iatrogenic causes, affecting the sympathetic neurons anywhere along its path [3]. Localization of the insult is the key to management. Sometimes it can present without anhidrosis, which is known as partial Horner’s syndrome [4] and indicates a pathology specifically beyond the superior cervical ganglion after the fibers have branched off. One of the more important causes that should be considered is carotid dissection [5], particularly in young adults less than 45 years of age, where it can account for 10-20% of all ischemic stroke cases [6] and is particularly relevant to this case.

## Case presentation

A 34-year-old white British female was normally fit and well with no significant medical history (aside from significant alcohol use, 56 units per week for several years). There was no use of medications aside from desogestrel 75 µg once daily as a contraceptive. She attended the Frimley Park Hospital A&E with a headache that started five days ago while she was on a two-week trip to the United States, where she was swimming and diving into pools. The headache was on her return and of sudden onset. It was located on the left side, radiating from her left eye to the scalp and dull in character, with a slight improvement in intensity compared to when it had started. She also had scalp tenderness, and three days ago, she noticed that her left eyelid was drooping. No other visual symptoms, rashes, neurological, chest, abdominal, or urinary symptoms were noted. The only positive family history was her grandfather having a transient ischemic attack at 56 years of age.

On examination, her vitals were stable. She was noted to have mild tenderness on her left scalp. Her left eyelid drooped compared to the right, and her left pupil was also smaller than the right, with poor accommodation, although a normal light reflex. She also had a mild horizontal nystagmus, which did not appear significant. Otherwise, her cranial nerves, cerebellar, motor, and sensory examinations were normal. There was no loss of sweating, suggesting partial Horner’s syndrome. Her initial blood findings are presented in Table 1.

**Table 1:** Routine blood findings on presentation. Unremarkable findings, although MCV was noted to be on the higher side. eGFR = estimated glomerular filtration rate; CRP = C-reactive protein; ESR = erythrocyte sedimentation rate; MCV = mean corpuscular volume; WBC = white blood cell; VBG = venous blood gas

Test name	Result	Reference range
Sodium	137	133–146 mmol/L
Potassium	4.1	3.5–5.3 mmol/L
Urea	2.2	2.5–7.8 mmol/L
Creatinine	61	49–90 µmol/L
eGFR result/1.73m² (EPI)	>90	90–120 mL/minute
CRP	2.7	0–5 mg/L
ESR	8	0–22 mm/hour
Hemoglobin	146	115–165 g/L
MCV	101	75–105 fL
WBC	8.8	4–11 × 10⁹/L
Platelets	296	150–450 × 10⁹/L
pH (VBG)	7.390	7.32–7.43
pCO_2_ (VBG)	5.80	4.6–6.0 kPa
Bicarbonate (VBG)	24.2	23–29 mmol/L
Lactate (VBG)	1.0	0.6–2.5 mmol/L

**Figure 1:**
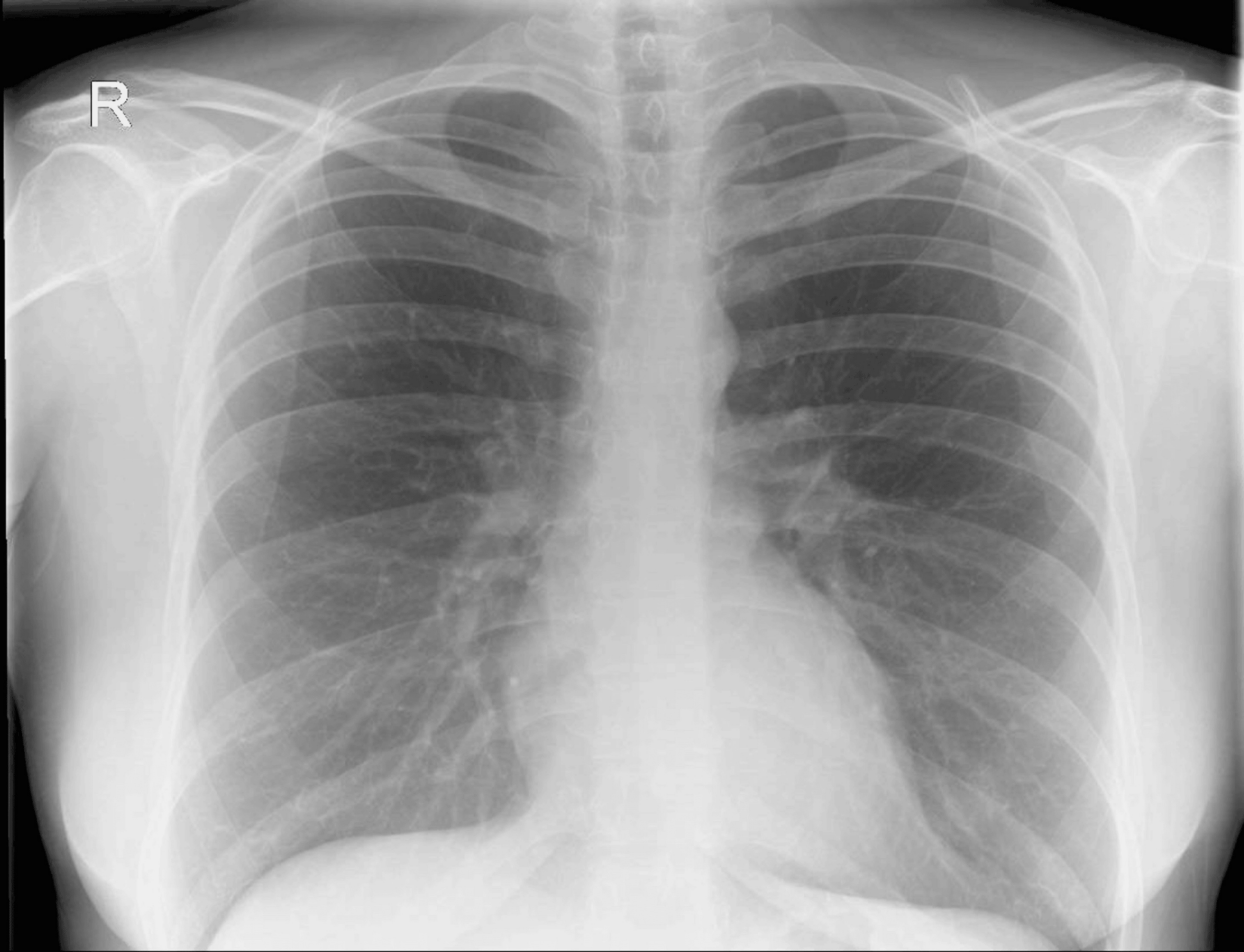
Chest X-ray posteroanterior view with an unremarkable appearance and clear apices, making a Pancoast tumor very unlikely.

A chest X-ray was performed to rule out a Pancoast tumor, which was unremarkable, as shown in Figure 1.

**Figure 2:**
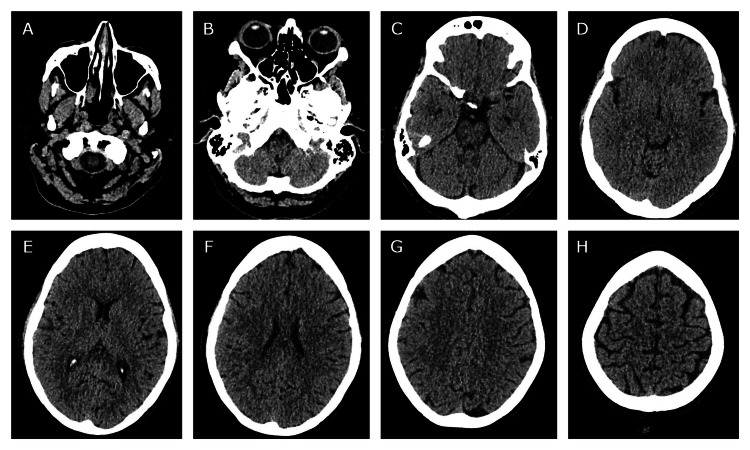
Multiple axial views of a non-contrast CT of the head from the skull base to the vertex in sequential images with no abnormality seen, particularly in the brainstem (A-D).

A CT of the head was also performed to rule out a brainstem stroke, which was also unremarkable, as shown in Figures 2A-2H.

**Figure 3:**
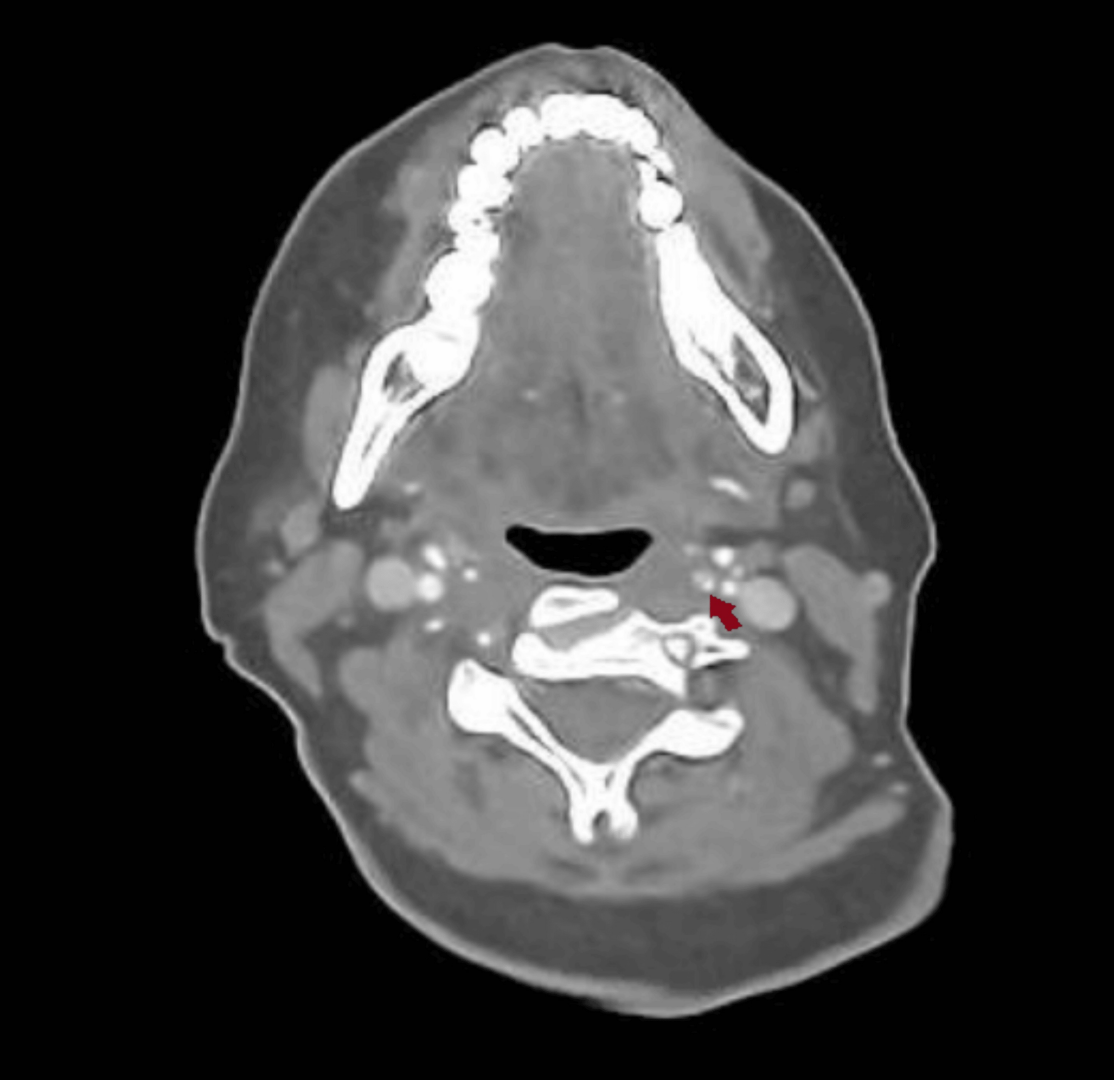
Axial CT carotid angiogram showing a narrow left internal carotid artery segment at the origin.

**Figure 4:**
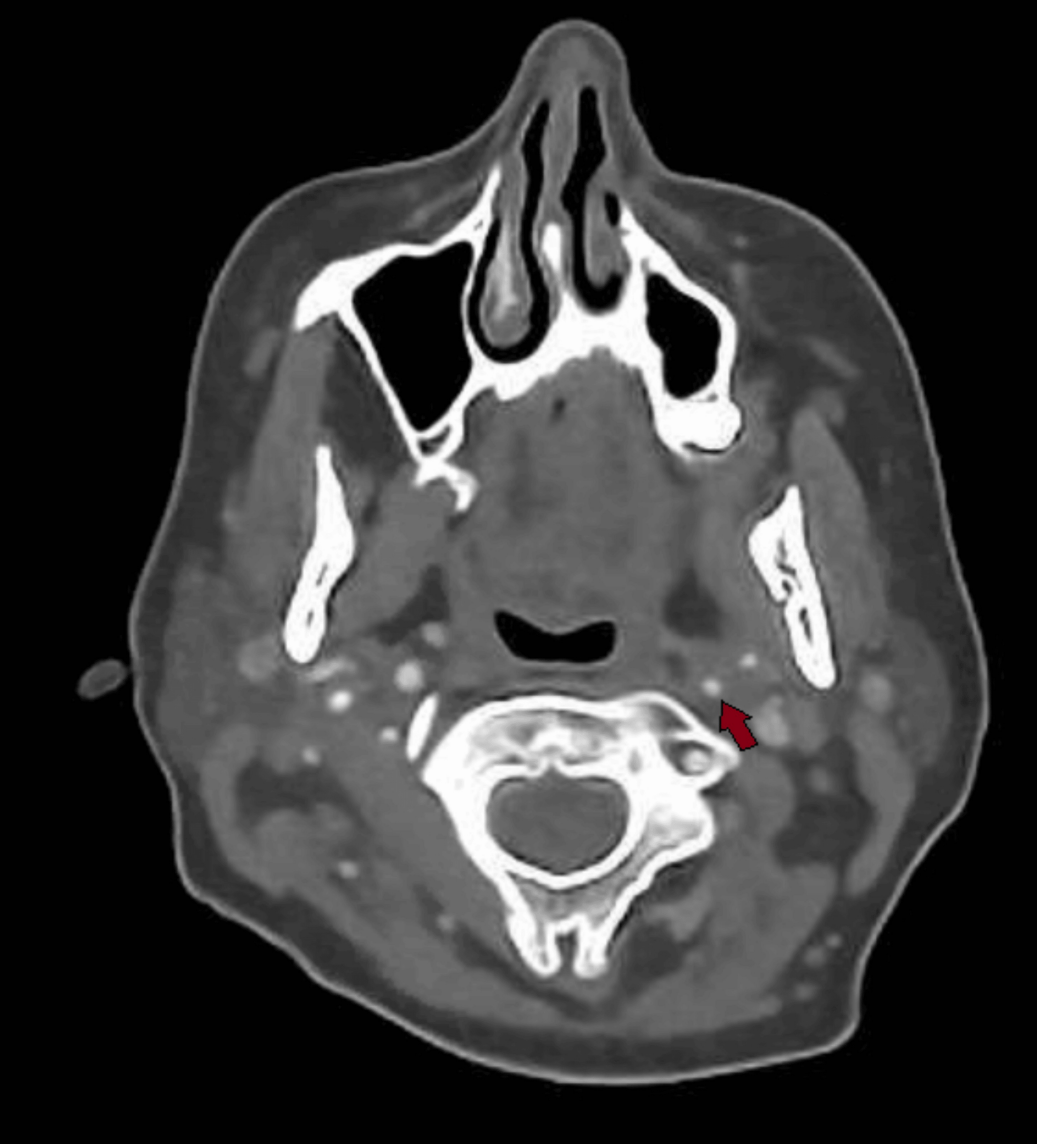
Axial CT carotid angiogram showing the left extracranial internal carotid artery with poor luminal filling seen.

**Figure 5:**
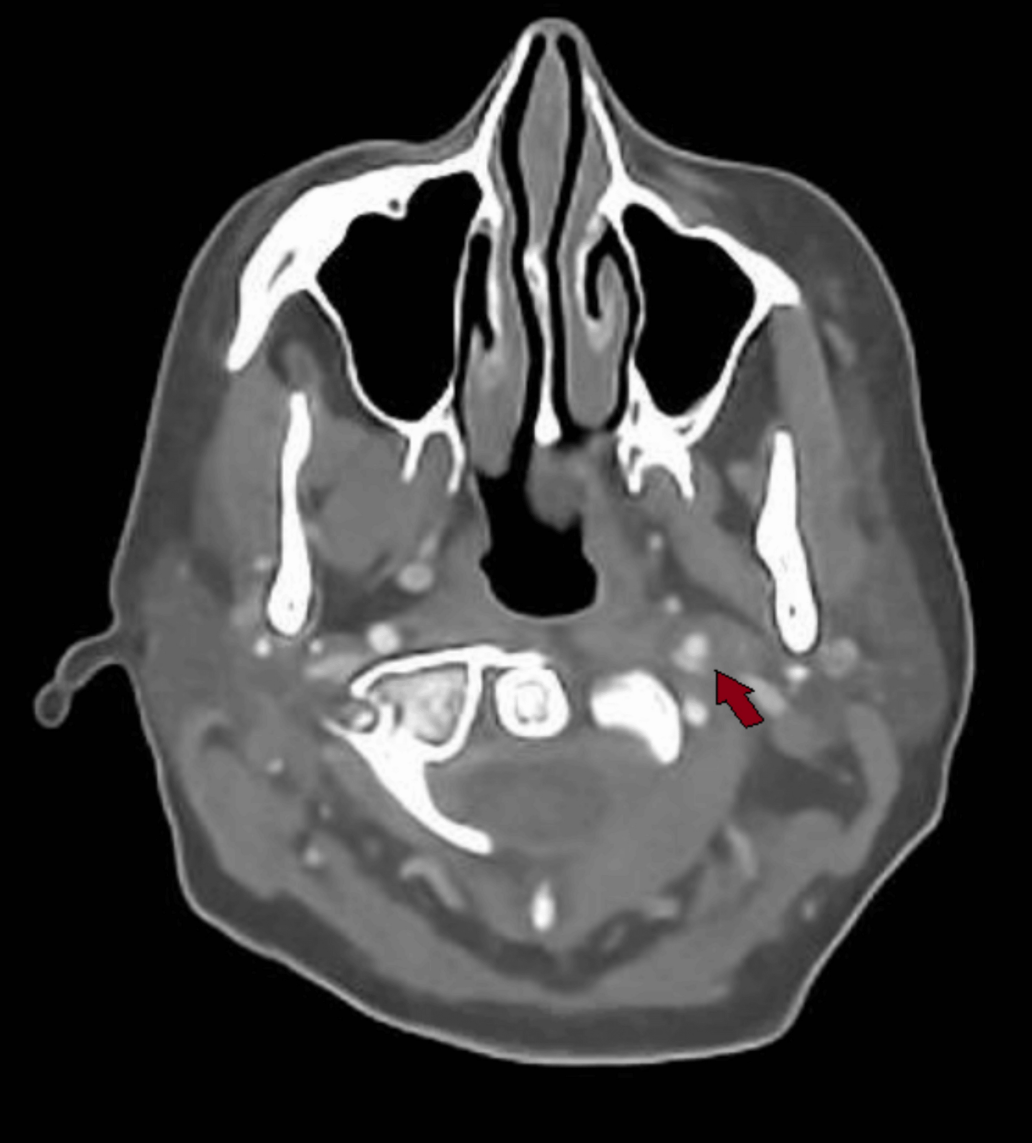
Axial CT carotid angiogram demonstrating a left extracranial internal carotid artery dissection flap with a false lumen.

**Figure 6:**
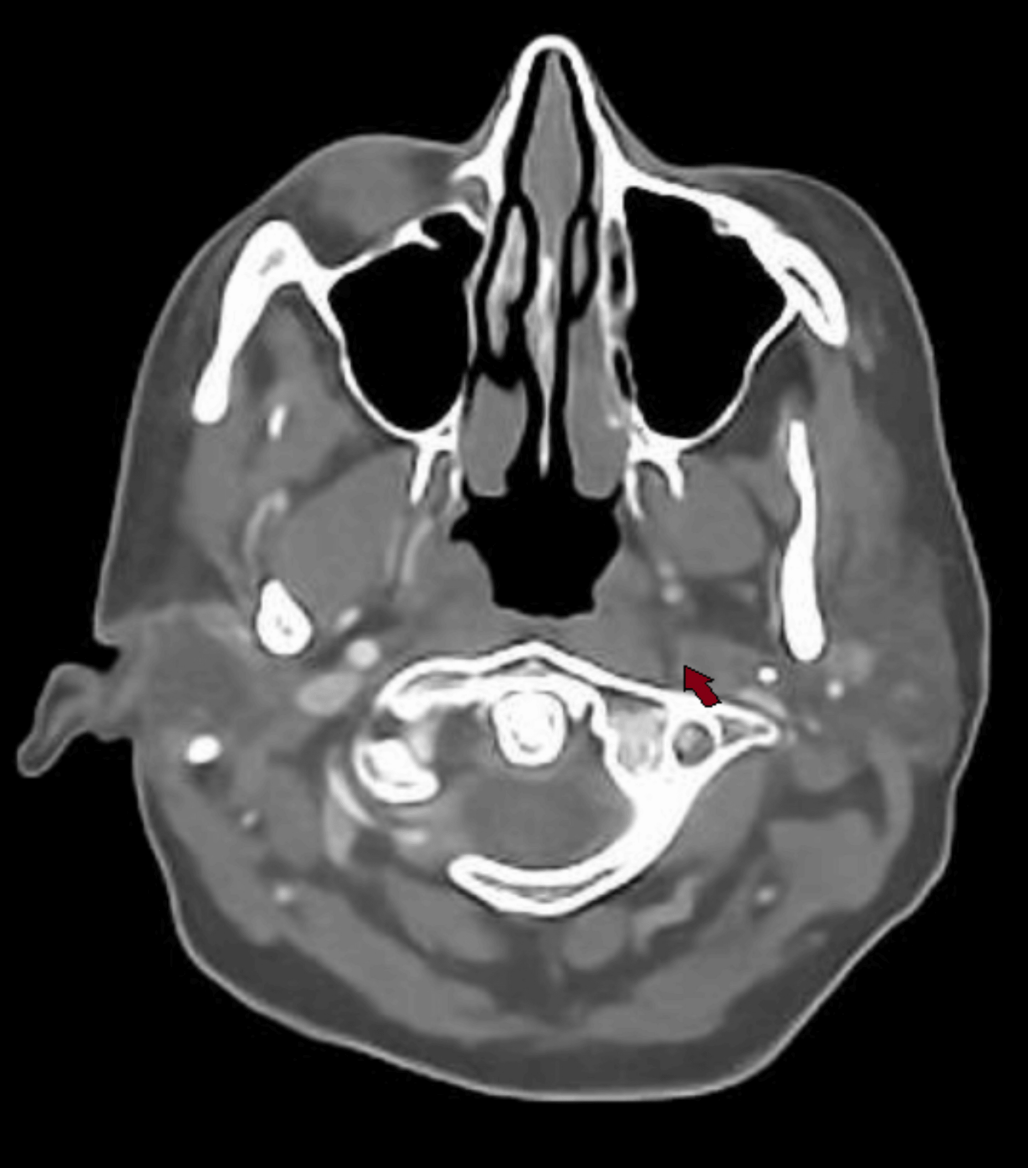
Axial CT carotid angiogram showing a lack of contrast flow in the left internal carotid artery beyond the dissection.

Due to her partial Horner’s syndrome and the suspicion of a carotid artery pathology causing it, a CT angiogram of the aortic arch and carotids was ordered, which showed an extracranial left internal carotid artery dissection and a short segment of very little luminal contrast before entering the carotid canal around C1/2, suggestive of a subtotal occlusion of the left internal carotid artery. This can be seen in sequential images from Figures 3-6, starting from the narrow left internal carotid artery at the origin and going up to and beyond the dissection with a resulting reduced contrast flow.

As a result, she was loaded with aspirin 300 mg, and her case was discussed with the stroke consultant, who accepted her under the stroke team, advising to load with clopidogrel 300 mg and start dual antiplatelet therapy the following day to stabilize the dissection and reduce the risk of thromboembolic complications. Her routine stroke blood findings are displayed in Table 2.

**Table 2:** Routine stroke blood findings showing a folate deficiency and slightly raised ferritin and ALT (not significant as it was also raised in her previous blood examination in 2022). TSH = thyroid-stimulating hormone; HDL = high-density lipoprotein; PT = prothrombin time; APTT = activated partial thromboplastin time

Test name	Result	Reference range
Total bilirubin	19	0–20 µmol/L
Alkaline phosphatase	80	30–130 U/L
Alanine transaminase	80	0–55 U/L
Albumin	40	35–50 g/L
Phosphate	1.02	0.8–1.5 mmol/L
Adjusted calcium	2.44	2.2–2.6 mmol/L
TSH	2.25	0.34–4.94 mIU/L
Cholesterol	4.5	<5 mmol/L
Non-HDL cholesterol	2.3	<4 mmol/L
HDL cholesterol	2.19	1.2–5 mmol/L
Cholesterol:HDL ratio	2.1	<4
Ferritin	311	30–250 µg/L
Prothrombin time	11.7	10.5–14.7 seconds
PT ratio	0.97	0.87–1.21
APTT	30.4	26.4–35.9 seconds
APTT ratio	0.98	0.85–1.15
Vitamin B12	348	200–900 ng/L
Folate	2.2	3–20.5 ug/L
HbA1c	29	20–41 mmol/mol

On a stroke consultant review the following day, the cause was still uncertain, with trauma, use of hormone therapy, and family history being among the possible causes. A young person’s stroke screen was requested (Table 3), followed by discharge on dual antiplatelet therapy and a plan for a repeat outpatient CT angiogram of the aortic arch and carotids in three months to check for resolution. A follow-up in the stroke clinic was also organized to monitor her blood and symptoms. On review, her symptoms appeared to have improved.

**Table 3:** Young person stroke screen result. The main finding is mild hyperhomocysteinemia, which is often seen in a folate deficiency.

Test name	Result	Reference range
Homocysteine	25.8	4.4–13.6 µmol/L
Anticardiolipin IgG antibody	1.6	0–9.9 GPLU/mL
Anticardiolipin IgM antibody	12.0	0–9.9 GPLU/mL
Beta 2 glycoprotein 1 IgG antibody	2	0–6 U/mL
Beta 2 glycoprotein 1 IgM antibody	< 2	0–10 U/mL
Myeloperoxidase antibody	< 0.2	0–3.4 U/mL
Proteinase 3 antibody	< 0.6	0–1.9 U/mL
DBS alpha-galactosidase - lower end of normal range	15.2	6.3–47.0 pmol/punch/hour
DBS alpha-galactosidase with inhibition	14.0	7.3–37.0 pmol/punch/hour

She was started on folate replacement with a plan to repeat her homocysteine levels two months after the folate levels had normalized. She was also advised to continue her dual antiplatelet therapy for at least three months until her follow-up CT angiogram, after which a further decision would be made regarding stopping or extending her antiplatelets by the stroke and neuroradiology multidisciplinary team, depending on the degree of resolution of her dissection.

## Discussion

Due to the length of the oculosympathetic pathway, Horner’s syndrome can be difficult to localize. One of the major clues in the presentation of our patient was the absence of anhidrosis, referred to as partial Horner’s syndrome [4], which suggests compression of the sympathetic nerve fibers in the third-order neuron distal to the carotid bifurcation, where the fibers split, sparing the sudomotor nerves, which resulted in intact hidrosis. She also had a headache, which is fairly common in association with Horner’s syndrome secondary to carotid dissection, being the initial presentation in more than half (58.5%) of cases and reported overall in nearly three-quarters (74%) of these patients, according to a case series involving 65 patients [7]. The same study also showed that the headaches were unilateral to the dissection in nearly four-fifths (79%) of cases, and could last from anywhere between one hour to 30 days, with a median time of five days. Almost a third of cases (31%) had a presentation with painful Horner’s syndrome similar to this case.

A CT of the head and chest X-ray was performed initially to rule out a brainstem stroke and Pancoast tumor; however, both of these are more likely to affect the first or second-order neurons, and based on her clinical presentation, the key investigation was the CT angiogram [8], which yielded the extracranial left internal carotid artery dissection and subtotal occlusion of the canal which was responsible for the symptoms. In addition to radiological tests, pharmacological tests are also used diagnostically to help localize the lesion, in particular cocaine, hydroxyamphetamine, and apraclonidine [9]. However, given the acute setting and risk of spurious results associated with these tests, as well as the presence of an obvious partial Horner’s syndrome suggesting a post-ganglionic pathology possibly related to the carotids, neuroimaging was deemed more appropriate and was expedited [10]. These tests are, however, more useful where radiological imaging might not be readily available or the lesion is more difficult to localize based on clinical assessment, and should still be considered.

Once the diagnosis was established and she was loaded on antiplatelets, the next challenge was determining the cause of her dissection. Carotid dissections are usually spontaneous or caused by trauma [11], which was a possibility given her recent travel and engaging in some light watersports, although there was no significant event reported. She was also known to be on hormonal therapy; however, while there is limited evidence of dissection in the cervical artery as per a recent study [12], the literature available for this is still scant. Some studies have suggested a reduction in carotid intima media thickness secondary to hormonal therapy [13]; however, this is only significant above 55 years, which does not fit with our patient. Family history was another possibility, although there was no history of dissection, and the only significant aspect was her grandfather having a transient ischaemic attack, but not a stroke, at 56 years of age. Additionally, according to a large study with nearly 2,000 participants, a family history of cervical artery dissection was found to be a very rare association, and the cause was multifactorial in the vast majority [14].

In addition to the above possibilities, her alcohol intake was also considered a risk factor, although no significant link has been identified between carotid wall thickness and alcohol consumption, according to a study [15], and light-to-moderate intake may even be associated with a reduced risk of mortality compared to non-drinkers [16]. However, her significant alcohol intake also appeared to have contributed to her having low folate levels, further resulting in mild hyperhomocysteinemia due to impaired re-methylation to methionine, both of which have been associated with an increased risk of cervical arterial dissections [17]. Hyperhomocysteinemia has been associated with damage to the arterial wall layers [18], even if just mildly elevated, as in this case, which could likely be a contributory factor to her dissection. While there is no direct treatment, folic acid supplementation could potentially reduce the risk of future episodes, which has also been suggested in a study that shows reduced progression of carotid intima-media thickness secondary to folate supplementation [19]. Folate supplementation has also proven to be beneficial for stroke prevention as a whole [20]. Thus, her folate deficiency and resulting mild hyperhomocysteinemia were thought to be the most significant contributory factors to her dissection, possibly reducing her threshold to trauma or resulting in an atraumatic dissection. This highlights the importance of a full stroke metabolic workup, especially in a younger patient with an unclear cause of dissection, as prompt management is likely to be of benefit, and identifying and correcting these deficiencies might reduce the risk of potential vascular events in the future.

## Conclusions

This case illustrates how a presentation of headache and unilateral ptosis can point toward a serious vascular insult such as carotid artery dissection. This is even more important in younger patients, under 45 years of age, who have a higher risk of carotid dissection in relation to ischemic stroke when compared to their elderly counterparts. Additionally, the absence of anhidrosis in her presentation provided a useful bedside clue that aided in localizing the lesion and led to prompt vascular imaging and early initiation of antiplatelet therapy. This is a useful lesson for clinicians, especially as the severity may increase if not identified early. A high degree of suspicion may be required in certain cases. The other significant learning here is the importance of identifying the potential risk factors and performing a comprehensive stroke workup. While her recent activity, alcohol use, and hormone therapy might have played a role, her folate deficiency and resulting mild hyperhomocysteinemia represent the most significant and potentially correctable risk factors that likely contributed to the arterial dissection. According to the literature, it appears that identifying and correcting reversible contributors such as folate deficiency can reduce the risk of these events, improve long-term outcomes, and reduce the risk of disability.
